# Dynamic Study on Water State and Water Migration during Gluten–Starch Model Dough Development under Different Gluten Protein Contents

**DOI:** 10.3390/foods13070996

**Published:** 2024-03-25

**Authors:** Haoxuan Ye, Yingquan Zhang, Lei Wang, Jinfu Ban, Yimin Wei, Fanghui Fan, Boli Guo

**Affiliations:** 1 Institute of Food Science and Technology, Chinese Academy of Agricultural Sciences/Comprehensive Utilization Laboratory of Cereal and Oil Processing, Ministry of Agriculture and Rural, Beijing 100193, China; yehaoxuan2021@email.szu.edu.cn (H.Y.); zhangyingquan@caas.cn (Y.Z.); weiyimin36@126.com (Y.W.); 2 Department of Food Science and Engineering, College of Chemistry and Environmental Engineering, Shenzhen University, Shenzhen 518060, China; 3 Institute of Western Agriculture, The Chinese Academy of Agricultural Sciences, Changji 831100, China; 4 Shijiazhuang Academy of Agricultural and Forestry Sciences, Shijiazhuang 050041, China; sjznkybjf@163.com

**Keywords:** dough development, water mobility, gluten, *LF-NMR*, secondary structure

## Abstract

Mixing is crucial for dough quality. The gluten content influences water migration in dough development and properties, leading to quality changes in dough-based products. Understanding how the gluten protein content influences water migration during dough development is necessary for dough processing. A compound flour with different gluten protein contents (*GPCs*, 10–26%, *w*/*w*) was used to study the dough farinograph parameters and water migration during dough development. According to the farinograph test of the gluten–starch model dough, the *GPC* increases the water absorption and the strength of the dough. Water migration was determined via low-field nuclear magnetic resonance (*LF-NMR*). With the increase in *GPC*, the gluten protein increases the binding ability of strongly bound water and promotes the transformation of weakly bound water. However, inappropriate GPC (10% and 26%, *w*/*w*) results in the release of free water, which is caused by damage to the gluten network according to the microstructure result. Moreover, the changes in proteins’ secondary structures are related to the migration of weakly bound water. Therefore, weakly bound water plays an important role in dough development. Overall, these results provide a theoretical basis for the optimization of dough processing.

## 1. Introduction

The mixing process is an important step in determining the quality of dough-based products. A dough is a complex system with interactions among proteins, starches, and water after mixing. The effect of mixing steps mainly involves the mixing of components, the hydration of components, and the formation of gluten networks during dough development [1]. Complex mixing processing introduces a question about quality control in the flour industry. Therefore, further research on dough development is necessary to control dough quality. After mixing, the dough creates a continuous gluten network, and the gluten network decides the rheology and processing adaptability of the dough. Starch granules embed in the continuous gluten network, and protein creates the viscoelastic properties of dough [2]. However, an insufficient mixing process may lead to an incompact dough and damage to the gluten network. In contrast, too extensive of a mixing time will depolymerize the glutenin macropolymer of dough and damage the gluten network [3]. Therefore, gluten is important for dough mixing and forming a gluten network. The effect of gluten on the mixing process is necessary.

Gluten protein is a major component of flour, which is important for forming a gluten network. Yang et al. [4] have shown that the presence of gluten is directly related to dough quality. Therefore, the effect of gluten protein on dough mixing is important. During mixing, the gluten network is formed by a disulfide bond, hydrogen bond, and hydrophobic bond between protein and water. The water provides a lot of hydrogen bonds for the protein to create a strong network in the dough. During mixing, the noncovalent bonds between proteins are weakened and the polymerized proteins’ molecular size decreases, while ω-gliadin is aggregated with other proteins through noncovalent bonds [5]. Noncovalent interactions of proteins help to stabilize the gluten network, but these bonds may affect the intersection of the gluten network after deformation [6]. Therefore, the hydration state of the protein is directly related to the linkage and the structure of the protein. Ortolan et al. [7] revealed protein characteristics in a gluten network and provided a comprehensive explanation of changes in protein structure in a gluten network. However, the knowledge of how gluten affects water migration is still lacking, which is important for dough processing. The variation in gluten protein content (*GPC*) in a dough may lead to a stronger or weaker interaction in the flour composition and affect the dough’s quality.

The development of the gluten network is highly dependent on the hydration process. Water provides a lot of hydrogen bonds to form a gluten network. The protein–protein interactions enhance the binding strength between the protein–water hydrogen bond and protein–protein hydrogen bond during the hydration process [8]. Water can be classified as strongly bound water, weakly bound water, and free water by its binding ability. The different water states play different roles in determining dough properties. Weakly bound water and free water are usually used as plasticizers in dough, while strongly bound water is typically utilized as a crosslinking agent between dough components [9]. The water determines the fluidity and swelling properties of wheat proteins, allowing them to respond to mechanical stress [10,11]. Bosmans et al. (2023) [12] studied the water state of different dough models (including starch–water, gluten–water, and flour–water models); however, the different development steps of dough had different hydration levels and led to changes in dough quality. It is necessary to discover the water migration process in the mixing steps and how water migration is influenced by the gluten content. Such knowledge can provide an optimal water level to improve dough quality.

Low-field nuclear magnetic resonance (*LF-NMR*) is widely used to detect diverse water distribution information through the proton relaxation behavior [13]. The transverse relaxation time (*T*_2_) and corresponding peak area (*A*_2_) collected by *LF-NMR* can be used to explain the water-binding ability and the water content, respectively. For a long time, *LF-NMR* has been successfully used to quantify the water state in different foods [14]. Further, a lot of research has studied the influence of processing or ingredients on the water mobility of dough products using *LF-NMR* [15,16]. However, the influence of *GPCs* on dough during mixing is still unclear. Therefore, *LF-NMR* was used to determine water migration during mixing with different *GPCs* in this study.

Herein, the compound flour consisting of wheat gluten and starch was used, and the wheat gluten proportion was used to adjust the *GPC.* The farinograph parameter of the dough with different *GPCs* was measured, and we studied the water migration process during the dough development process under different *GPCs*. The secondary structure of protein and the water states were used to explain the changes in properties during the mixing process. Further, the dough microstructure in the mixing process was observed by scanning electron microscopy. This study discovered the influence of the protein on the strength of dough, and the mechanism of water migration during dough mixing was revealed. These data can provide a theoretical basis for the mixing process and quality control of wheat flour and wheat-based products.

## 2. Materials and Methods

### 2.1. Preparation of the Gluten–Starch Model Dough

The gluten–starch model dough consists of different proportions of starch and wheat gluten (Guanxian Xinrui Industrial Co., Ltd., Liaocheng, China). The proportion of wheat gluten is used to adjust the *GPC* (10%, 14%, 18%, 22%, and 26%). The protein content of wheat gluten and wheat starch are 76.6% and 0.24%, respectively. The water content of compound flour is as follows: 10% *GPC* flour: 12.50%; 14% *GPC* flour: 11.80%; 18% *GPC* flour: 11.90%; 22% *GPC* flour: 11.60%; and 26% *GPC* flour: 11.20%, *w*/*w*.

### 2.2. Farinograph Test and the Sampling Point

According to AACCI Approved Method 54–21.02 (2010), a farinograph (E-type, Brabender, Duisburg, Germany) was used to mix the dough and determine the dough farinograph properties of the compound flours. During dough mixing, the thermostat of the farinograph was set at 30 °C. The amount of water added to the dough was adjusted to achieve a maximum consistency of 500 BU. The water absorption (WA), dough development time (DDT), dough stability time (DST), degree of softening (DS), and farinograph quality number (FQN) were recorded.

**Figure 1:**
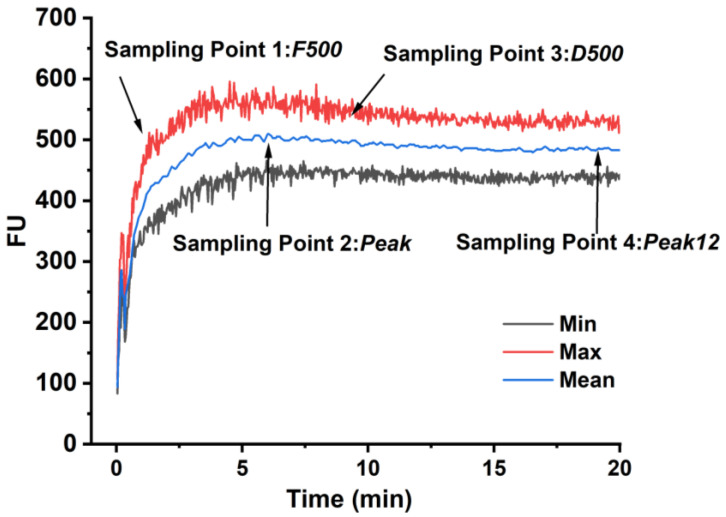
Sampling point in the mixing process of dough.

According to farinograph curves obtained in the previous step, four typical sampling points of dough were collected in this research and described in Figure 1. Sampling point 1: when the maximum dough consistency reaches 500 BU for the first time (*F500*). Sampling point 2: when the dough reaches peak consistency (*Peak*). Sampling point 3: when the maximum dough consistency drops to 500 BU (*D500*). Sampling point 4: continued mixing 12 min after the dough consistency reaches its peak (*Peak12*). Parts of the dough were sampled at the sampling point for subsequent experiments. Moreover, about 20 g of dough was immediately taken and frozen in liquid nitrogen, then dried using a vacuum freeze-dryer (1-2LD PLUS, Chirst, Hanover, Niedersachsen, Germany) for 48 h. Each sample was sampled from a different dough, as the farinograph instrument could not restart once sampling was stopped; however, each dough was made in replicate by following the same material and processing method.

### 2.3. Low-Field Nuclear Magnetic Resonance (LF-NMR)

About 2 g of fresh dough was wrapped in Teflon film and sealed separately to prevent moisture loss during testing. The transverse relaxation time (*T*_2_) of the dough sample in different mixing stages was measured by the *LF-NMR* system with a permanent magnet of 0.5 T (NMI20-030H-I; Niumag Analytical Instrument Corporation, Suzhou, China). The proton resonance frequency of this *LF-NMR* was 21 MHz at 32 ± 0.01 °C. To achieve the Carr–Purcell–Meiboom–Gill (*CPMG*) decay signals, a radio frequency coil with a 5 mm diameter was used. The echo time was set to 0.101 ms and the number of sampling points was 121,204. The echo number was set to 12,000 and the scan reiterations were set to 64. Each experiment was performed in triplicate. Multi Exp Inv analysis software (v1.0, Niumag Analytical Instrument Corporation, Suzhou, China) calculated the *CPMG* decay curves that fit the distributed multiexponential. The relaxation data were analyzed with the instrument inbuilt software algorithm to improve the multiexponential fitting analysis. The relaxation time for each process was determined by their peak positions and the accumulative integral was used to calculate the area of each peak.

### 2.4. Secondary Structure of Dough

The 5 mg freeze-dried dough sample was weighed and ground evenly with potassium bromide (KBr) at a ratio of 1:100. Then, the sample was tableted for Fourier infrared spectrum scanning (Nicolet™ iS™ 5 FTIR, Thermo Scientific, Waltham, MA, USA). The spectrum of the 1600–1700 cm^−1^ segment was taken for Gaussian deconvolution and second derivative, and the percentage of each secondary structure was calculated. Each sample was repeated 3 times.

### 2.5. Scanning Electron Microscopy of the Dough Microstructure

The freeze-dried dough samples were cut off with pliers. Cut samples with a flat surface were selected and fixed on the sample platform. The dough samples were then placed under a scanning electron microscope (SU8010, Hitachi, Tokyo, Japan) at 1000 times magnification, observed, and photographed.

### 2.6. Statistical Analysis

SPSS 21.0 was used for statistical analysis of the data collected from the experiments. One-way ANOVA was used to analyze the difference in treatment conditions. Duncan’s multiple comparisons were used to analyze the significance of the differences. The significance level was *p* < 0.05.

## 3. Results and Discussion

### 3.1. Effects of Gluten Content on the Farinograph Parameters

**Figure 2:**
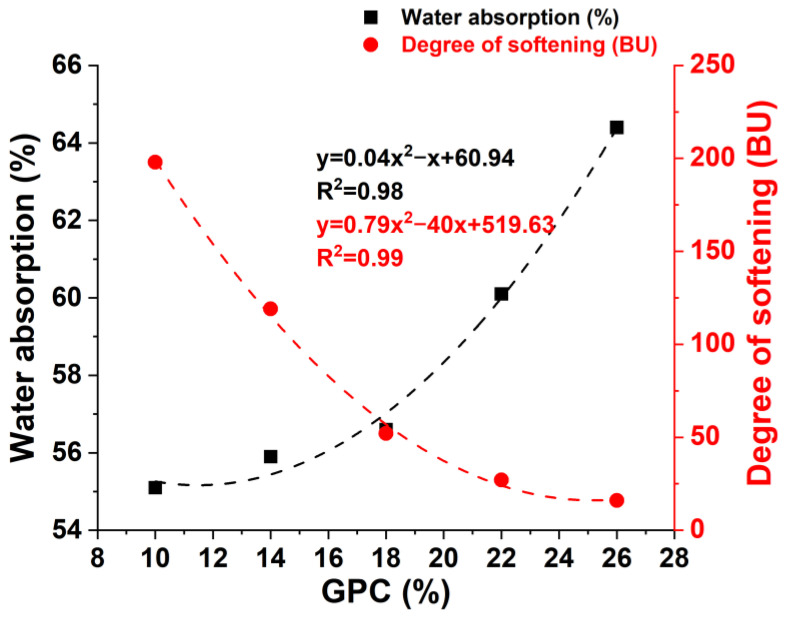
The water absorption and degree of softening of the compound flour.

As the *GPC* of the dough increased from 10% to 26%, the development time increased from 0.5 min to 6.0 min and the stability time showed the same trend. The water absorption increased from 55.1% to 64.4% and the degree of softening decreased from 198 BU to 16 BU (Table 1). This suggests that increasing *GPC* enhances the water-absorption ability and the dough strength, which is consistent with previous studies [17,18]. Moreover, the increase in the development and stability time also indicates a stronger gluten network of dough. This means that a higher *GPC* needs more time to establish an interaction between the components and water in the dough. The water in the dough can be divided into starch-bound water and protein-bound water; the protein-bound water is considered to serve a dominant role, and the water cross-links to the protein and starch in the dough [19]. The starch binds water through the hydrogen bond of the hydrophilic group, e.g., the water-hydroxyl group bond [12]. The highest-mobility water is present in an easily mobile environment, e.g., a pore in the network. The lowest-mobility water is trapped in the amylopectin and amylose helices as crystallized water [20]. The higher water absorption of the dough is due to the proteins being able to bind and trap more water, and the presence of hydrogen bonds in water may contribute to forming a more stable protein–starch network structure. The increase in *GPC* enhances the dough’s strength because the gluten network behaves like an elastic solid and provides elasticity and strength to prevent the collapse of the dough [21]. The proteins unfold to form a continuous viscoelastic network during the mixing process, which includes disulfide bond organization, hydrogen bond arrangement, hydrophobic interaction, and tangles, and produce new polymerized proteins [22]. Thus, the addition of proteins may help form polymeric proteins by creating a cross-linking effect, reducing the degree of softening (Figure 2). The polynomial fitting of the correlation between *GPC* and water absorption of dough has a higher regression coefficient than liner fitting because starch and other ingredients also have significant effects on the capacity of the dough to absorb water.

### 3.2. The Present State of Water (T_2_) during Dough Development

**Figure 3:**
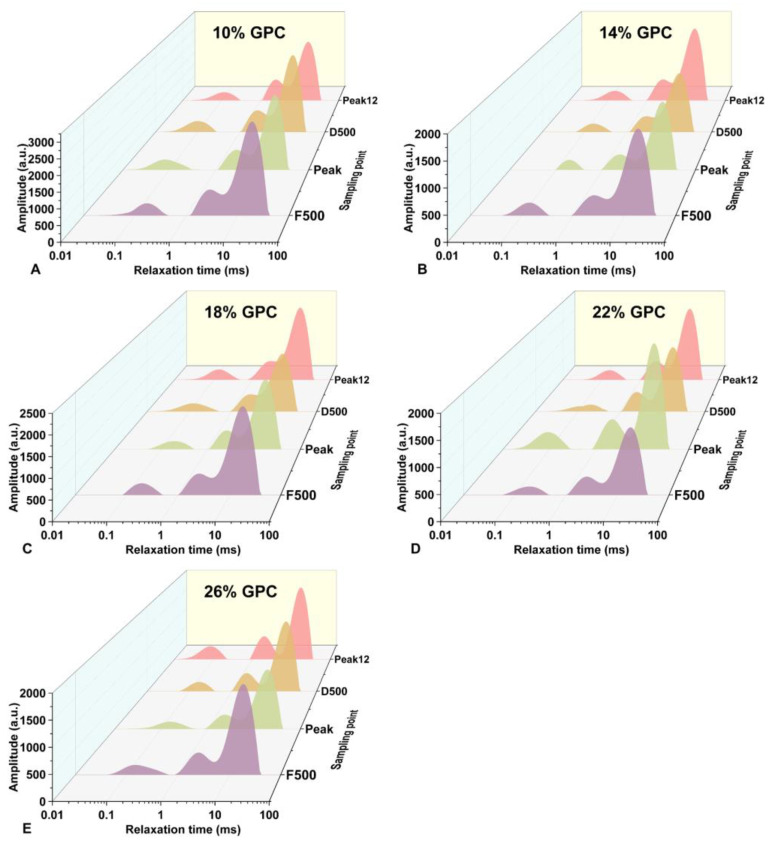
(**A**–**E**) The transverse relaxation changes of water during dough mixing with different *GPCs* (10–26%, *w*/*w*).

The water’s transverse relaxation time can serve as an indicator of binding ability in the dough, and a shorter *T*_2_ indicates a higher binding ability and lower mobility of water. *T*_21_ is the relaxation time of strongly bound water, which is tightly bound to starch or gluten protein, and *T*_22_ is the relaxation time of weakly bound water; the fluidity of weakly bound water is between strongly bound water and free water. *T*_23_ is the relaxation time of free water, which is adsorbed on the surface of the dough [23,24]. The transverse relaxation time (*T*_2_) spectra of the dough at different *GPCs* are shown in Figure 3. *T*_2_ all show a decreasing trend during development. This indicates that the interaction between water and the dough becomes stronger during development.

#### 3.2.1. The Relaxation Time of Strongly Bound Water (*T*_21_) during Dough Development

**Figure 4:**
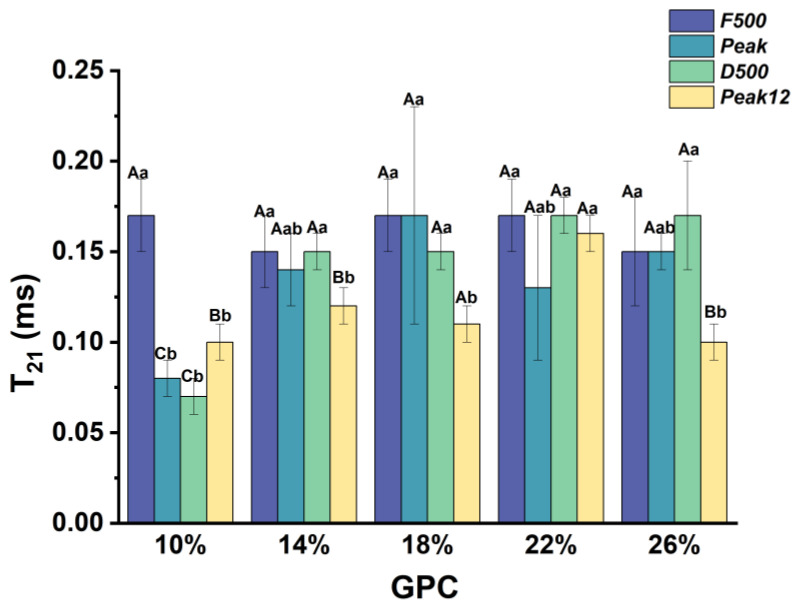
*T*_21_ changes during dough development under different *GPCs.* Data are expressed as mean ± standard deviation (*n* ≥ 3), the difference in *GPC* in the same sampling point is expressed in lowercase letters (*p* < 0.05), and the difference in different sampling points in the same *GPC* is expressed in uppercase letters.

Figure 4 shows the variation in *T*_21_ in the dough sample during dough development. *T*_21_ of the sample decreased first and then increased at 10% *GPC*. The *T*_21_ value is high at the *F500* point, which suggests that the strongly bound water is relaxed in this mixing stage. Defour et al. (2023) [25] also found a similar phenomenon in an undeveloped mixing dough. This can be explained by the fact that the dough had not yet established a tight gluten network in the early mixing stage and the water had not yet been tightly combined with proteins [16]. With the development of the dough, more water is combined with the polar groups of the side chains of protein amino acids and reduces the *T*_21_ [26]. However, the changes in *T*_21_ all show similar change trends in high *GPC* (>10%, *w*/*w*). *T*_21_ shows no significant changes at the first three sampling points and then decreases in *Peak12* (>10% *GPC*, *w*/*w*). Because more *GPC* participated in the gluten network, the higher mechanical energy required to break the relation between the protein makes the strongly bound water exhibit low mobility. With dough development, *T*_21_ decreased due to the excessive mixing time, allowing water to completely combine with proteins.

We found that the increase in *GPC* mainly affected the *T*_21_ in *Peak* and *Peak12* points. Compared with *T*_21_ of the 10% *GPC* sample, *T*_21_ of *Peak* and *D500* points increased significantly, indicating that the increase in *GPC* could increase the *T*_21_. This suggests increased *GPC* weakens the interaction between strongly bound water and the dough system. The increase in gluten proportion reduces the starch content, which makes the gluten network lack enough starch to fill the network, and the loose gluten network increases the fluidity of strongly bound water. The mechanical stress of stirring might spread along the polymer chain, and then the shear-induced hydrogen bond dissociation and reconstruction leads to strongly bound water migration [25].

#### 3.2.2. The Relaxation Time of Weakly Bound Water (*T*_22_) during Dough Development

**Figure 5:**
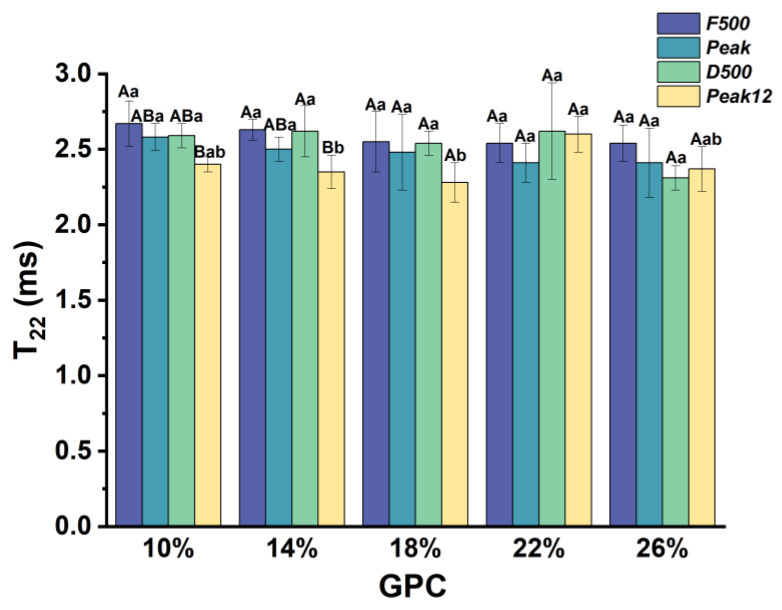
*T*_22_ changes during dough development under different *GPCs.* The difference in *GPC* in the same sampling point is expressed in lowercase letters (*p* < 0.05), and the difference in different sampling points in the same *GPC* is expressed in uppercase letters.

The relaxation time *T*_22_ of weakly bound water is shown in Figure 5. *T*_22_ decreased during development, especially at *Peak12*. This means that the interaction of weakly bound water became stronger during development. With a mixing time that was too long, the gluten network was depolymerized and the gluten network was damaged at *Peak12*. Partially weakly bound water was released and the remaining weakly bound water became tighter in the gluten network [25].

However, *T*_22_ shows no significant difference in the first three sampling points under different *GPCs*. This indicates that gluten protein only affects *T*_22_ of the *Peak12* point. Only *T*_22_ of 22% *GPC* is significantly higher than that of 14% and 18% *GPC*. This is because the samples with high protein content can leave more pores for weakly bound water in the gluten network.

#### 3.2.3. The Relaxation Time of Free Water (*T*_23_) during Dough Development

**Figure 6:**
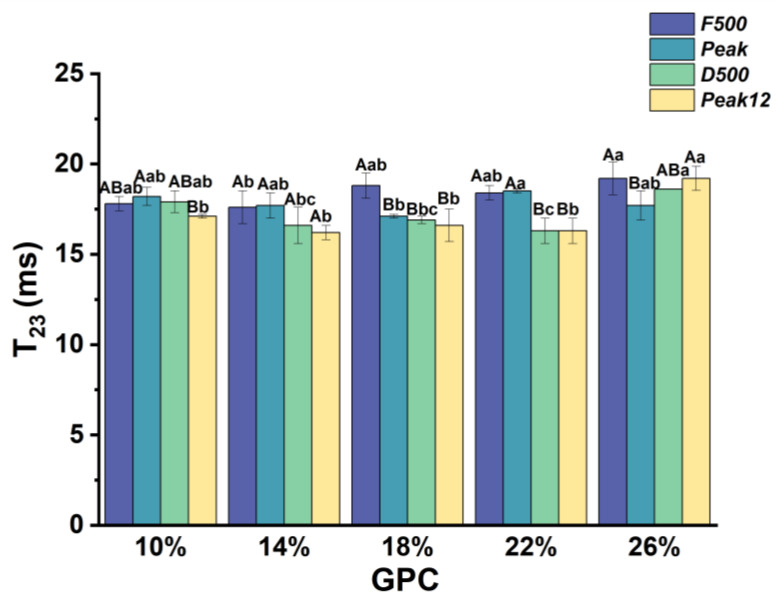
*T*_23_ changes during dough development under different *GPCs.* The difference in *GPC* in the same sampling point is expressed in lowercase letters (*p* < 0.05), and the difference in different sampling points in the same *GPC* is expressed in uppercase letters.

*T*_23_ represents free water with a high degree of freedom. *T*_23_ of the dough varied from 16.24 to 19.2 ms, depending on the *GPC* and sampling point (Figure 6). *T*_23_ of the dough with 10–22% *GPC* showed a decreasing trend with dough development. However, *T*_23_ of the dough with 26% *GPC* first decreased and then increased with dough development. This indicates that the participation of excess protein releases free water under extended mixing. This may be due to excessive mechanical energy intake during the stirring process, which causes the disintegration of dough with a permanent structure and releases free water [10].

*T*_23_ of 26% *GPC* is higher than 14% *GPC* at the *F500* point, and *T*_23_ of 22% *GPC* is higher than 18% *GPC* at the *Peak* point. Therefore, increasing *GPC* may increase the free water fluidity in the early mixing stage. At the *D500* point, we found that *T*_23_ first decreased and then increased with the increase in *GPC*. Therefore, an appropriate *GPC* would decrease the *T*_23_ and help to stabilize the dough. The appropriate ratio of starch and gluten protein leads to more covalent and noncovalent bonds between the hydrophilic groups on the surface of starch particles and the gluten network, thus making the dough structure more compact [27,28]. However, inadequate or excessive *GPC* enhances free water fluidity. Meanwhile, at excessive *GPC*, the insufficient starch filling makes the gluten network vulnerable to destruction, thus releasing free water. Moreover, Liu et al. (2023) [29] claimed that the strong hydrophilicity of starch may compete with some water molecules, which should be noted. Similarly, previous studies have shown that wheat granular starch can absorb between 39% and 87% of water by weight, damaged starch between 200% and 430%, and proteins between 114% and 215% [30]. Therefore, starch also influences the hydration ability of dough, which should be considered in dough processing.

### 3.3. The Water Ratio (A_2_%) Changes during Dough Development

**Figure 7:**
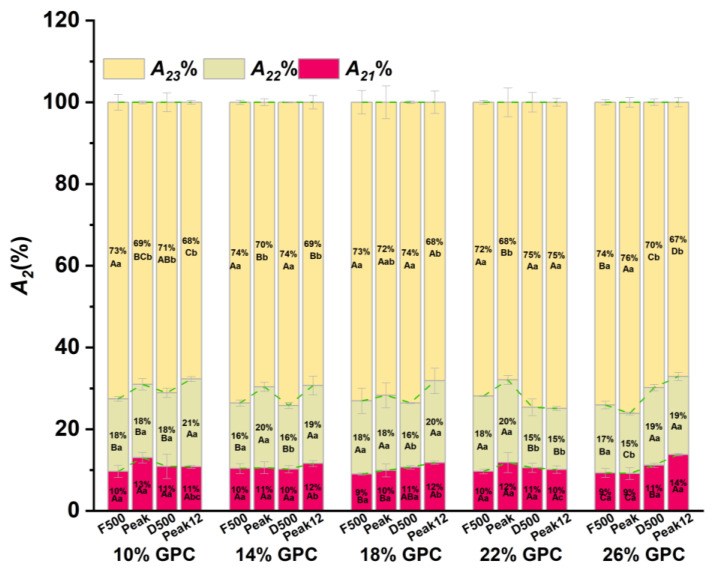
*A*_2_% changes during dough development under different *GPCs.* The difference in *GPC* in the same sampling point is expressed in lowercase letters (*p* < 0.05), and the difference in different sampling points in the same *GPC* is expressed in uppercase letters.

Figure 7 shows the corresponding peak areas of bound water (*A*_21_%), weakly bound water (*A*_22_%), and free water (*A*_23_%) in the dough at different *GPCs* during mixing. The total peak integral area represents the water content of the sample. Due to the complete sealing of the sample, moisture loss is not considered in the dough’s mixing process. Therefore, the dough with the same *GPC* had the same water content at different sampling points. Differences in the *GPC* and sampling points showed a significant influence on the peak integral areas of water (*p* < 0.05). The *A*_2_*%* exhibits the same change trend in low *GPC* (≤18%, *w*/*w*). However, the 22% and 26% *GPCs* exhibit entirely different changes in *A*_2_%.

#### 3.3.1. The Water Ratio of Strongly Bound Water during *A*_21_% Dough Development

As seen in Figure 7, strongly bound water comprises the smallest proportion of water in the dough. The *A*_21_% of 10%, 14%, and 22% *GPC* did not significantly change during dough development (Figure 7). However, the *A*_21_% of 18% and 26% increased during dough development. This indicates that the increase in *GPC* can increase the *A*_21_% of the sample. The appropriate addition of *GPC* enables the encapsulation of starch particles and the formation of a dense gluten network. When the GPC is increased to 26%, the higher protein content provides a lot of water binding sites, e.g., the polar groups of the side chains of protein amino acids, thereby increasing the presence of strongly bound water and *A*_21_% [6,26].

No significant difference in *A*_21_% between different *GPCs* was found at the first three sampling points. This indicates that proteins do not affect the strongly bound water content of the underdeveloped dough. However, at *Peak12*, the *A*_21_% of 26% *GPC* was significantly higher than other sampling points. This can be explained as the increase in hydrogen bond interaction between water and gluten due to the increase in proteins so that the strong bound water content in the structure increases [31]. It should be noted that the *A*_21_% of 22% *GPC* shows no significant difference with 10% *GPC*; this is due to the mechanical damage in the dough influencing the binding of water. The changes in the strength of the dough should consider the influence of the filling of starch and the strengthening effect of gluten protein.

#### 3.3.2. The Water Ratio of Weakly Bound Water (*A*_22_%) during Dough Development

There is a significant difference in *A*_22_*%* at different sampling points and *GPCs* but the changes in *A*_22_% are irregular (Figure 7). Therefore, it is hard to explain the mechanism of dough development induced by *A*_22_%. The changes in *A*_22_% are particularly affected by gluten’s three-dimensional structure, which provides a cavity to contain weakly bound water [15]. At the *F500* point, *A*_22_% exhibited no significant changes among the different *GPCs*. At the *Peak* point, the 26% *GPC* had the lowest *A*_22_%. A lower *A*_22_% at *D500* was found in the 14–22% *GPC* sample compared to 10% *GPC* and 26% *GPC*. At the *Peak12* point, the lowest *A*_22_% was found in 22% *GPC*. Therefore, increasing the *GPC* leads to a decrease in *A*_22_% among different *GPCs*.

#### 3.3.3. The Water Ratio of Free Water (*A*_23_%) during Dough Development

Free water is the major portion of water in the dough and determines its rheology and texture. The *A*_23_% of the dough showed a similar trend of change in the *GPC*, less than 26%, and *A*_23_% first decreased and then increased and then decreased again (Figure 7). During dough development, free water and bound water transform each other through the polymerization and depolymerization of the gluten network of dough. The decrease in *A*_23_% at the peak points indicates that the protein aggregation of the dough and the free water transforms into bound water. Then, water is released at *D500* due to structural damage caused by long-time stirring, and then part of the free water content is reduced at *Peak12* due to the repolymerization of part of the protein network structure [15,25]. However, at 26% *GPC*, *A*_23_% first increases at the *Peak* point and then decreases after. This indicates that the changes in *A*_23_% are affected by a high *GPC*.

*A*_23_% at the *F500* point shows no significant difference between different *GPCs*, the same as *A*_21_% and *A*_22_%, indicating that the *GPC* has little effect on the water content when the dough is not fully developed (Figure 7). At the *Peak* point, the free water transformed to strongly bound water in 10% *GPC* but, with the increase in *GPC* (14%, 22%, and 26%), the free water transformed to weakly bound water simultaneously. However, at the *D500* point, the strongly bound water in 10% *GPC* was converted to free water and, with the increase in *GPC* to 22%, the weakly bound water was also converted to free water. Therefore, the appropriate *GPC* can help the weakly bound water to participate in the water mobility of the dough, which is conducive to the dough’s stability. When the dough reached the *Peak12* point, the changes in *A*_23_% of high *GPC* (≥22%, *w*/*w*) showed different trends. The *A*_2_% of 22% *GPC* showed no significant change from point *D500* to *Peak12*, while 26% *GPC* showed that the free water was converted to strongly bound water (Figure 7). The 22% *GPC* tends to stabilize after a long stirring time because the higher strength of the dough makes water migration difficult. Meanwhile, the *A*_23_% of the 26% *GPC* sample continued to decrease because more gluten polymer was destroyed over a longer mixing time, releasing free water, and exposing more water-binding sites. This led to an increase in strongly bound water. The influence of protein on water is very complex. In the process of dough mixing, the mechanical depolymerization of gluten protein is accompanied by the breakage of covalent bonds [32] and the weakening of electrostatic interaction and the hydrogen bond [33], and the competition of starch also affects water migration in the dough. Therefore, an extensive or insufficient *GPC* also damages dough development; the resistance of the dough to mechanical force and the influence of dough damage on water migration should be considered simultaneously.

### 3.4. The Changes in Protein Secondary Structure during Dough Development

The secondary structure of the dough is listed in Table 2. Both the mixing process and *GPC* have a significant influence on the secondary structure (*p* < 0.05). With increasing *GPC* and development, the secondary structure reaches an equilibrium value. The β-turn increased as the intramolecular β-sheet declined, showing that the protein network became gradually organized and stable. Because β-turn is the preferred secondary structure after gluten hydration, increasing the concentration of β-turn decreases the concentration of the β-sheet and random coil structure [33]. According to the “loop-train” model, the increase in β-turn indicates the formation of a “loop” structure, which provides the viscoelasticity properties of dough [34]. The increase in the intermolecular β-sheet structure is attributed to the influence of hydrogen bonds between glutenin molecules, which contribute to forming larger loop structures when the intermolecular β-sheet is further hydrated, which appears as protein aggregation [35,36]. Because α-helix is the primary skeleton of proteins and is responsible for their ordered structure, a rise in α-helix during mixing may indicate that the structure is becoming more organized. Polypeptide chains with α-helix structures have higher strength and flexibility [2]. When the *GPC* was increased to 22%, it was discovered that extra protein had no significant effect on the secondary structure of the protein, which could be due to the increased *GPC* promoting cross-linking between proteins in the dough and causing the protein of the dough to reach a relatively stable state quickly. Moreover, less starch was present and the proteins could compete for more water for adequate hydration, reducing differences in secondary structure between different sample points with high *GPC* (≥22%, *w*/*w*). The increase in α-helix during mixing means the structure becomes more ordered.

### 3.5. Correlation Analysis among Water States and the Protein Secondary Structure

**Figure 8:**
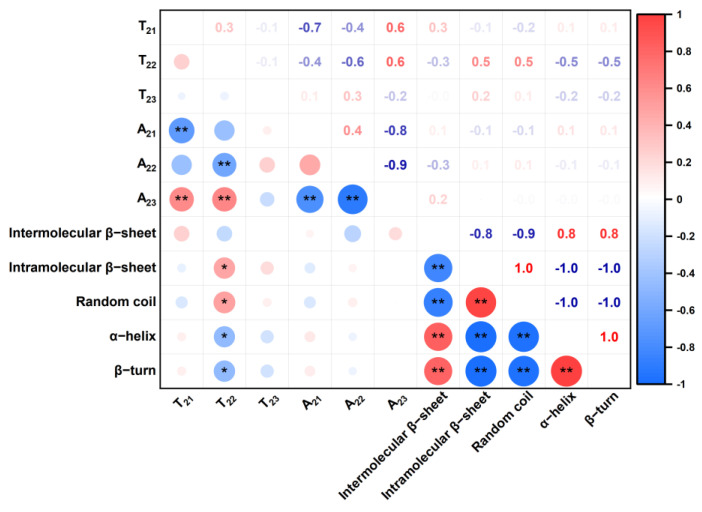
The relationship between secondary structure and water states. * indicates significantly related (*p* < 0.05) and ** indicates extremely significantly related (*p* < 0.01).

The correction relation analysis between water states and protein secondary structure is described in Figure 8. The *T*_22_ of weakly bound water was significantly related to the protein secondary structure (*p* < 0.05), while there was no significant difference in *A*_22_ with different *GPCs*. This means that the changes in protein secondary structure mainly influence the binding ability of weakly bound water.

α-helix is an ordered dough structure; the significant negative correlation between α-helix and *T*_22_ indicates that the ordered structure can limit the weakly bound water migration. The opposite explanation can describe the relationship between a random coil and *T*_22_. β-turn also has a negative correlation with *T*_22_. Ortolan et al. [7] claimed that the β-turn can form a spiral structure and enhance protein–solution interaction, which is why β-turn would decrease *T*_22_. Moreover, the intramolecular β-sheet is used to describe the aggregation of protein. The intramolecular β-sheet is positively corrected to the *T*_22_; this may be due to the expulsion of water from gluten polymerization and the increase in the weakly bound water’s mobility [12]. Therefore, the secondary structure is related to weakly bound water; this suggests that the weakly bound water influences the gluten network during dough development.

### 3.6. Network Structure Changes during Dough Development

**Figure 9:**
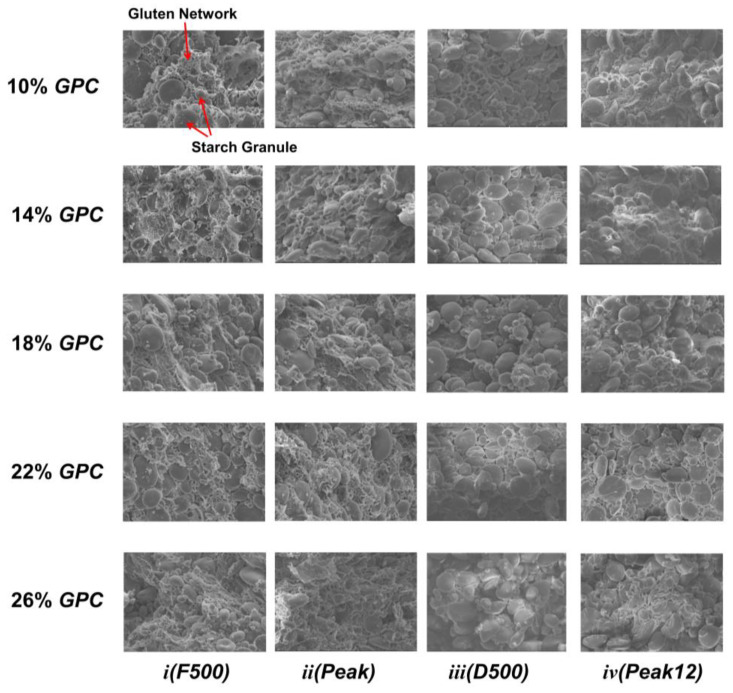
The 1000 ×magnification microscopic images of dough with 10–26% *GPCs* during mixing. (***i***) *F500*. (***ii***) *Peak*. (***iii***) *D500*. (***iv***) *Peak12*.

The *SEM* shows the morphology structure with different *GPCs* in Figure 9. In the mixing process, water migration and the change in dough morphology are two simultaneous dynamic equilibria. When the consistency of the dough reached 500 BU, the starch granules would absorb water, and the gluten protein form a network via the hydrogen bond provided by bound water in this stage. However, the network is loose and the starch easily peels off, as the composition does not allow for a compact interaction. The binding ability of water was relatively weaker. Moreover, Liu et al. (2023) [29] claimed the starch interaction is weak at this time. When the dough consistency reached the maximum, the gluten network was compact and the water was further bound with the composition (Figure 9(*i*)). At that moment, the binding capacity of water was stronger because gluten and starch were hydrated well [3]. At the *D500* point, the gluten network was completely developed. Starches were distributed evenly in the gluten network. Therefore, the bound water has the tightest binding force in this state, forming a homogeneous three-dimensional dough network. The *Peak12* point exhibits a damaged gluten network with many large pores inside. This phenomenon is consistent with a previous study, where the gluten network was torn and broke down at an extended mixing time [11]. A broken gluten network makes it hard to embed starch and bind water. At the same time, the water was released from the broken gluten network.

With the increase in *GPC*, the 26% *GPC* in *F500* showed smooth gluten (Figure 9(*iv*)). The lower starch granule exposure on the surface in 26% *GPC* and a high *GPC* help to establish a compact gluten network, which makes the bound water tighter as a result. However, the microstructure of the dough showed little difference in its *Peak* point. At the *D500* point, a bare gluten network in 10% *GPC,* the insufficient gluten protein experiences difficulty warping the starch and the long mixing time knocks off the starch. This is why *T*_21_ of 10% *GPC* at the *D500* point shows the lowest value compared to other groups. Moreover, at the *Peak12* point, we found a lot of protein pieces adherent with the starch granule in 26% *GPC*. This releases the free water and provides more water-binding sites for strongly bound water (Figure 9(*iv*)). The dough microstructure of 10% *GPC* at the *Peak12* point also showed a broken gluten network, which is due to a weak gluten network that is unable to resist the damage of mechanical force. What is different is that the 18% and 22% *GPCs* at the *Peak12* point can keep a smooth gluten network, which is consistent with the result of *A*_2_%. This indicates that over-mixing damages the gluten and part of the protein is broken, which releases the free water and increases *A*_23_% in 26% *GPC* (Figure 7). Therefore, changes in the morphology of the gluten network are accompanied by water migration.

## 4. Conclusions

In this study, gluten–starch model dough with different *GPCs* (10–26%, *w*/*w*) was examined. The increases in *GPC* significantly enhance water absorption and dough strength, requiring more time for dough development. During dough development, the increase in *GPC* decreases the *T*_21_ of dough and an inappropriate *GPC* (10% and 26%, *w*/*w*) increases the *T*_23_. The result of changes in *A*_2_% revealed that the *GPC* can help the weakly bound water to participate in water transformation, which is better for forming viscoelastic dough. Furthermore, changes in the secondary structure primarily involve a decrease in the intramolecular β-sheet to increase other secondary structures during the mixing process, leading to the dough becoming orderly and stable. An increase in α-helix is related to the binding of weakly bound water. The result of *SEM* revealed that an inappropriate *GPC* (10% and 26%, *w*/*w*) damages the gluten network, which is consistent with the changes in *A*_2_%. In summary, the dough development process is a complex process influenced by mechanical forces and interactions between components. Therefore, the *GPC* should be controlled at an appropriate level to balance the strength of the dough and the integrity of the gluten network. These results significantly enhance our understanding of how the *GPC* influencing the mixing process of dough is crucial for the wheat-based product industry.

## Figures and Tables

**Table 1:** The farinograph parameters of compound dough with different *GPCs*.

*GPC* (%)	Water Absorption (%)	Development Time (min)	Stability Time (min)	The Degree of Softening (BU)	Farinograph Index (mm)
10	55.1	0.5	0.5	198	7
14	55.9	0.5	0.8	119	8
18	56.6	0.9	8.1	52	18
22	60.1	3.5	21.5	27	111
26	64.4	6.0	36.1	16	165

**Table 2:** Secondary structure of dough with different *GPCs* during development.

GPC (%)	Sampling Point	Intermolecular β-Sheet (%)	Intramolecular β-Sheet (%)	α-Helix (%)	Ramdon Coil (%)	β-Turn (%)
10	*F500*	6.03 ± 0.08 Bb	31.23 ± 0.17 Aa	20.74 ± 0.06 Aa	18.55 ± 0.18 Bb	23.46 ± 0.09 Bb
	*Peak*	5.88 ± 0.04 Bb	30.93 ± 0.08 Aa	20.79 ± 0.05 Aa	18.72 ± 0.06 Bb	23.67 ± 0.07 Bb
	*D500*	5.66 ± 0.03 Bb	30.66 ± 0.15 Aa	20.8 ± 0.18 Aa	18.7 ± 0.14 Bb	24.19 ± 0.15 Bb
	*Peak12*	7.66 ± 0.19 Aa	13.89 ± 0.3 Ba	19.93 ± 0.15 Ba	23.7 ± 0.3 Aa	34.82 ± 0.09 Aa
14	*F500*	6.01 ± 0.06 Bb	30.85 ± 0.46 Aa	20.53 ± 0.32 Aa	18.72 ± 0.32 Bb	23.9 ± 0.55 Bb
	*Peak*	5.99 ± 0.05 Bb	30.38 ± 0.02 Aa	20.61 ± 0.05 Aa	18.8 ± 0.09 Bb	24.21 ± 0.1 Bb
	*D500*	8.22 ± 0.15 Aa	14.44 ± 0.22 Bb	19.72 ± 0.12 Ba	23.45 ± 0.18 Aa	34.17 ± 0.12 Aa
	*Peak12*	7.99 ± 0.03 Aa	14.46 ± 0.03 Ba	19.64 ± 0.04 Ba	23.35 ± 0.04 Aa	34.57 ± 0.06 Aa
18	*F500*	5.46 ± 0.07 Bb	30.09 ± 0.08 Aa	20.74 ± 0.08 Aa	18.83 ± 0.09 Bb	24.87 ± 0.05 Bb
	*Peak*	5.98 ± 0.13 Bb	30.3 ± 0.11 Aa	20.42 ± 0.07 Aa	18.7 ± 0.12 Bb	24.61 ± 0.07 Bb
	*D500*	7.89 ± 0.04 Aa	13.61 ± 0.08 Bb	19.51 ± 0.07 Ba	23.67 ± 0.04 Aa	35.31 ± 0.16 Aa
	*Peak12*	8.01 ± 0.23 Aa	13.8 ± 0.22 Ba	19.68 ± 0.09 Ba	23.55 ± 0.22 Aa	34.96 ± 0.16 Aa
22	*F500*	8.19 ± 0.2 Aa	14.11 ± 0.25 Ab	19.63 ± 0.04 Aa	23.61 ± 0.16 Aa	34.46 ± 0.32 Aa
	*Peak*	8.29 ± 0.04 Aa	14.11 ± 0.06 Ab	19.33 ± 0.03 Aa	23.49 ± 0.04 Aa	34.77 ± 0.11 Aa
	*D500*	8.1 ± 0.13 Aa	13.89 ± 0.2 Ab	19.61 ± 0.08 Aa	23.65 ± 0.05 Aa	34.75 ± 0.43 Aa
	*Peak12*	7.85 ± 0.11 Aa	13.6 ± 0.06 Aa	19.6 ± 0.15 Aa	23.7 ± 0.09 Aa	35.24 ± 0.1 Aa
26	*F500*	8.12 ± 0.16 Aa	13.85 ± 0.28 Ab	19.46 ± 0.13 Aa	23.51 ± 0.12 Aa	35.05 ± 0.35 Aa
	*Peak*	8.21 ± 0.21 Aa	14.07 ± 0.22 Ab	19.52 ± 0.15 Aa	23.59 ± 0.17 Aa	34.61 ± 0.26 Aa
	*D500*	8.14 ± 0.22 Aa	13.95 ± 0.24 Ab	19.52 ± 0.15 Aa	23.61 ± 0.1 Aa	34.78 ± 0.32 Aa
	*Peak12*	8.15 ± 0.26 Aa	13.97 ± 0.34 Aa	19.49 ± 0.13 Aa	23.57 ± 0.11 Aa	34.82 ± 0.51 Aa

The difference in *GPC* in the same sampling point is expressed in lowercase letters (*p* < 0.05), and the difference in different sampling points in the same *GPC* is expressed in uppercase letters.

## Data Availability

The original contributions presented in the study are included in the article, further inquiries can be directed to the corresponding authors.

## References

[1] Dufour M., Chaunier L., Lourdin D., Réguerre A.L., Hugon F., Dugué A., Kansou K., Saulnier S., Della Valle G. (2024). Unravelling the relationships between wheat dough extensional properties, gluten network and water distribution. Food Hydrocoll..

[2] Wang Y., Tacer-Caba Z., Immonen M., Kemell M., Varis J.J., Jian C., Maina N.H. (2022). Understanding the influence of in situ produced dextran on wheat dough baking performance: Maturograph, biaxial extension, and dynamic mechanical thermal analysis. Food Hydrocoll..

[3] Dhal S., Anis A., Shaikh H.M., Alhamidi A., Pal K. (2023). Effect of Mixing Time on Properties of Whole Wheat Flour-Based Cookie Doughs and Cookies. Foods.

[4] Yang J., Chen L., Guo B., Zhang B., Zhang Y., Li M. (2022). Elucidation of rheological properties of frozen non-fermented dough with different thawing treatments: The view from protein structure and water mobility. J. Cereal Sci..

[5] Wang X., Appels R., Zhang X., Bekes F., Diepeveen D., Ma W., Hu X., Islam S. (2020). Solubility variation of wheat dough proteins: A practical way to track protein behaviors in dough processing. Food Chem..

[6] Ortolan F., Corrêa G.P., da Cunha R.L., Steel C.J. (2017). Rheological properties of vital wheat glutens with water or sodium chloride. LWT.

[7] Ortolan F., Urbano K., Netto F.M., Steel C.J. (2022). Chemical and structural characteristics of proteins of non-vital and vital wheat glutens. Food Hydrocoll..

[8] Jia R., Zhang M., Yang T., Ma M., Sun Q., Li M. (2022). Evolution of the morphological, structural, and molecular properties of gluten protein in dough with different hydration levels during mixing. Food Chem. X.

[9] Liu S., Liu Q., Li X., Obadi M., Jiang S., Li S., Bin X. (2021). Effects of dough resting time on the development of gluten network in different sheeting directions and the textural properties of noodle dough. LWT.

[10] Baudouin F., Nogueira T.L., van Der Mijnsbrugge A., Frederix S., Redl A., Morel M.H. (2020). Mechanochemical activation of gluten network development during dough mixing. J. Food Eng..

[11] Abang Zaidel D.N., Chin N.L., Yusof Y.A., Abdul Rahman R., Karim R. (2009). Statistical modelling of gluten production by varying mixing time, salt and water levels during dough mixing. Int. J. Food Eng..

[12] Bosmans G.M., Lagrain B., Deleu L.J., Fierens E., Hills B.P., Delcour J.A. (2012). Assignments of proton populations in dough and bread using NMR relaxometry of starch, gluten, and flour model systems. J. Agr. Food Chem..

[13] Ling X., Tang N., Zhao B., Zhang Y., Guo B., Wei Y.M. (2020). Study on the water state, mobility and textural property of Chinese noodles during boiling. Int. J. Food Sci. Technol..

[14] Kamal T., Cheng S., Khan I.A., Nawab K., Zhang T., Song Y., Wang S., Nadeem M., Raiz M., Khan MA U. (2019). Potential uses of LF-NMR and MRI in the study of water dynamics and quality measurement of fruits and vegetables. J. Food Process..

[15] Yang Y., Guan E., Zhang T., Li M., Bian K. (2019). Influence of water addition methods on water mobility characterization and rheological properties of wheat flour dough. J. Cereal Sci..

[16] Liu H., Xing Y., Liu D., Yang Q., Xiao S., Fu Y., Wang X. (2023). Quality deterioration of frozen dough bread during terminal freezing and thawing: From the insight of moisture and starch properties. Food Biosci..

[17] Li M., Liu C., Zheng X., Hong J., Bian L., Li L. (2021). Interaction between A-type/B-type starch granules and gluten in dough during mixing. Food Chem..

[18] Sissons M.J., Soh H.N., Turner M.A. (2007). Role of gluten and its components in influencing durum wheat dough properties and spaghetti cooking quality. J. Sci. Food Agric..

[19] Gras P.W., Carpenter H.C., Anderssen R.S. (2020). Modelling the developmental rheology of wheat-flour dough using extension tests. J. Cereal Sci..

[20] Ritota M., Gianferri R., Bucci R., Brosio E. (2008). Proton NMR relaxation study of swelling and gelatinisation process in rice starch–water samples. Food Chem..

[21] Uthayakumaran S., Newberry M., Keentok M., Stoddard F.L., Bekes F. (2020). Basic rheology of bread dough with modified protein content and glutenin-to-gliadin ratios. Cereal Chem..

[22] Schurer F., Kieffer R., Wieser H., Koehler P. (2007). Effect of hydrostatic pressure and temperature on the chemical and functional properties of wheat gluten II. Studies on the influence of additives. J. Cereal Sci..

[23] Wang Y.H., Zhang Y.R., Yang Y.Y., Shen J.Q., Zhang Q.M., Zhang G.Z. (2022). Effect of wheat gluten addition on the texture, surface tackiness, protein structure, and sensory properties of frozen cooked noodles. LWT.

[24] Zhang S., Sun X., Xu X., Feng X., Wang Z., Meng L., Wu D., Tang X. (2022). Effects of soaking conditions on the quality and in vitro starch digestibility of extruded whole buckwheat noodles. J. Cereal Sci..

[25] Dufour M., Foucat L., Hugon F., Dugué A., Chiron H., Della Valle G., Saulnier L. (2023). Water mobility and microstructure of gluten network during dough mixing using TD NMR. Food Chem..

[26] Liu H., Wan L., Xiao S., Fu Y., Wang X. (2023). Changes in the physicochemical and protein distribution properties of dough with the wheat oligopeptide incorporation. Food Biosci..

[27] Yang Y.L., Guan E.Q., Zhang L.L., Pang J.Y., Li M.M., Bian K. (2021). Effects of vacuum degree, mixing speed, and water amount on the moisture distribution and rheological properties of wheat flour dough. J. Food Sci..

[28] Šćepanović P., Goudoulas T.B., Germann N. (2018). Numerical investigation of microstructural damage during kneading of wheat dough. Food Struct..

[29] Liu L., Hu X., Zou L. (2023). Wheat polysaccharides and gluten effect on water migration and structure in noodle doughs: An 1H LF-NMR study. J. Cereal Sci..

[30] Berton B., Scher J., Villieras F., Hardy J. (2020). Measurement of hydration capacity of wheat flour: Influence of composition and physical characteristics. Powder Technol..

[31] Guo L., Fang F., Zhang Y., Xu D., Jin Z., Xu X. (2021). Glutathione affects rheology and water distribution of wheat dough by changing gluten conformation and protein depolymerisation. Int. J. Food Sci. Technol..

[32] Wang X., Choi S.G., Kerr W.L. (2004). Water dynamics in white bread and starch gels as affected by water and gluten content. LWT.

[33] Iwaki S., Fu B.X., Hayakawa K. (2023). Behavior of protein aggregates via electrostatic interactions or hydrogen boynds during dough formation. J. Cereal Sci..

[34] Bock J.E., Damodaran S. (2013). Bran-induced changes in water structure and gluten conformation in model gluten dough studied by fourier transform infrared spectroscopy. Food Hydrocoll..

[35] Anjum F.M., Khan M.R., Din A., Saeed M., Pasha I., Arshad M.U. (2007). Wheat gluten: High molecular weight glutenin subunits—Structure, genetics, and relation to dough elasticity. J. Food Sci..

[36] Chen G., Ehmke L., Miller R., Faa P., Smith G., Li Y. (2018). Effect of sodium chloride and sodium bicarbonate on the physicochemical properties of soft wheat flour doughs and gluten polymerization. J. Agr. Food Chem..

